# A practical approach to the management of percutaneous coronary intervention complications

**DOI:** 10.3389/fcvm.2026.1760313

**Published:** 2026-02-05

**Authors:** Tanawat Attachaipanich, Hafeez Ul Hassan Virk, Muzamil Khawaja, Mahboob Alam, Ravi S. Hira, Jacob A. Doll, Chayakrit Krittanawong

**Affiliations:** 1 Department of Internal Medicine, University of Missouri-Kansas City School of Medicine, Kansas City, MO, United States; 2 Division of Cardiovascular Disease, Case Western Reserve University, Cleveland, OH, United States; 3 Department of Cardiology, Emory University, Atlanta, GA, United States; 4 Texas Heart Institute and Baylor College of Medicine, Houston, TX, United States; 5 Pulse Heart Institute and Multicare Health System, Tacoma, WA, United States; 6 Foundation for Health Care Quality, Seattle, WA, United States; 7 Cardiovascular Division, Department of Medicine, University of Washington, Seattle, WA, United States; 8 VA Puget Sound Health Care System, Seattle, WA, United States; 9 HumanX, Delaware, DE, United States

**Keywords:** ACS—ACS/NSTEMI, PCI, percutaneous coronary intervention (PCI), complex PCI, PCI complications

## Abstract

**Introduction:**

Despite advances in stent design, pharmacotherapy, and procedural techniques that have improved percutaneous coronary intervention (PCI) outcomes and reduced PCI-related complications, these events still occur and are associated with adverse outcomes. Moreover, complex PCI procedures, which predispose to increased risk of complications, are increasingly performed. Understanding risk factors, underlying mechanisms, evidence-based management, and preventive strategies are essential to optimize procedural outcomes. This review aims to summarize current evidence and highlight gaps in knowledge related to PCI-associated complications.

**Methods:**

This narrative review used a focused PubMed search through October 2025, prioritizing randomized trials, large observational studies, guidelines, and consensus statements.

**Results:**

Acute vessel closure, which most commonly results from dissection or thrombosis and perforation are frequently associated with hemodynamic compromise and increased procedural mortality. Device-related complications such as entrapment and fracture, although rare, can potentially lead to significant morbidity and mortality. Preventive strategies emphasize appropriate lesion preparation, proper device selection and sizing, gentle manipulation, and the use of adjunctive imaging modalities such as intravascular ultrasound and optical coherence tomography to minimize risk. Early recognition and prompt management of these complications are essential to decreased adverse outcomes of PCI in both short and long term. However, due to the low incidence of these events, current management strategies are largely based on case reports, observational studies, and expert consensus.

**Conclusions:**

Future large-scale studies and registry data, along with artificial intelligence-guided risk modeling are warranted to facilitate individualized prediction, enhance procedural safety, and advance precision management across preprocedural, intraprocedural and postprocedural phases.

## Graphical Abstract

**Figure:**
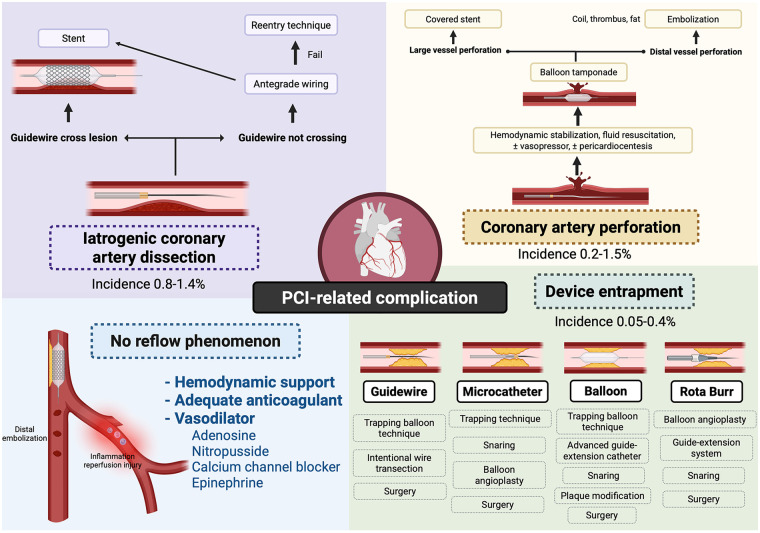
PCI-Related Complications and Management Strategies. This schematic summarizes major complications associated with percutaneous coronary intervention (PCI), including iatrogenic coronary artery dissection, coronary perforation, no-reflow phenomenon, and device entrapment. Created in BioRender. Attachaipanich T. (2026) https://BioRender.com/mpuf12a, licensed under Academic License.

## 1 Introduction

Percutaneous coronary intervention (PCI) has become one of the most frequently performed cardiovascular procedures, with over 600,000 PCI cases performed annually at more than 1,600 centers across the United States (1). Advances in stent design, procedural techniques, and the use of potent antithrombotic therapies have markedly improved clinical outcomes and reduced procedural complications (2). Despite these improvements, PCI-related complications still occur and remain clinically significant, contributing to increased morbidity and mortality (3). Furthermore, the rising prevalence of complex PCI procedures has been associated with a higher risk of adverse events (4). A comprehensive understanding of the underlying mechanisms, risk factors, management approaches, and preventive strategies is essential to minimize these complications and optimize patient outcomes. The aim of this review is to summarize current evidence on PCI-related complications, focusing on intraprocedural coronary-related complications, including acute vessel closure, coronary dissection, coronary perforation, device entrapment or fracture, stent loss, and air embolism. These include the underlying pathophysiologic mechanisms to evidence-based management and prevention strategies, with the goal of guiding clinical decision-making and improving PCI outcomes in contemporary practice. This narrative review was based on a focused literature search of PubMed from inception through October 2025 using terms related to PCI-related complications. Priority was given to randomized clinical trials, large observational studies, registry data, consensus statements, and contemporary guideline documents, with inclusion of relevant case series and expert opinion when higher-level evidence was limited.

## 2 Acute vessel closure

### 2.1 Description

Acute vessel closure refers to the abrupt cessation of anterograde coronary perfusion, leading to myocardial ischemia. In the NHLBI Dynamic Registry, an observational registry study, the incidence of acute vessel closure declined from approximately 3% during the balloon angioplasty era to 0.3% in the contemporary PCI era, largely due to advances in stent technology and potent antithrombotic therapy (5). Acute vessel closure may result from coronary dissection, thrombosis, distal embolization, side branch occlusion after stenting, vasospasm, pseudolesion formation, equipment entrapment, intramural hematoma, and no-reflow, with dissection and intracoronary thrombus being the predominant causes (6).

### 2.2 Risk factors

Risk factors for acute vessel closure include patient-related factors such as female sex, prior myocardial infarction (MI), presentation with acute MI, and high surgical risk (6). Angiographic and procedural factors include multivessel disease, eccentric or complex lesions, multiple lesions within the same vessel, long lesion length, and bifurcation or branch-point lesions (6).

### 2.3 Management strategies

Management of acute vessel closure involves maintaining wire access, promptly identifying the underlying mechanism, and performing targeted intervention based on identified mechanism, while maintaining hemodynamic stability (6). Understanding these mechanisms, particularly dissection and thrombosis, is essential for prevention, early recognition, and timely management.

## 3 Coronary artery dissection

### 3.1 Iatrogenic coronary artery dissection

#### 3.1.1 Description

Iatrogenic coronary artery dissection is a rare but serious complication of PCI and represents one of the major mechanisms of acute vessel closure. The reported incidence of iatrogenic coronary dissection during PCI ranges from 0.8% to 1.4% (7–9). Iatrogenic coronary artery dissection most commonly results from noncoaxial catheter or guide engagement, forceful contrast injection against a dampened pressure waveform, inadvertent deep seating of the guide catheter, or excessive manipulation of stiff guiding catheters (7–9). Aggressive advancement or withdrawal of interventional devices and the use of polymer-jacketed guidewires may further propagate a dissection flap. In addition, balloon rupture, particularly in the presence of a calcified lesion, can result in an aortocoronary dissection (7–9). Importantly, although mechanical injury typically initiates the dissection, subsequent contrast injections may propagate a hydraulic dissection, further extending the intramural hematoma and potentially leading to vessel occlusion (10).

#### 3.1.2 Risk factors

Patient-related factors include advanced age, female sex, and presentation with ACS (11). Angiographic risk factors include calcified, eccentric, long, or complex lesions (ACC/AHA type B or C), as well as vessel tortuosity (11). Procedural risk factors include a large balloon-to-artery ratio (>1.2), noncoaxial guide alignment, and deep seating of the catheter (11). Dissection is typically visualized on coronary angiography. Once a dissection is identified, further antegrade contrast injection should be avoided (10). Intravascular imaging, particularly IVUS, can facilitate accurate diagnosis and delineation of the dissection's extent (10).

In addition, underlying connective tissue disorders and pathogenic collagen or extracellular matrix gene variants have been associated with an increased risk of spontaneous coronary artery dissection, and syndromic conditions such as Loeys-Dietz syndrome have been reported as rare causes of SCAD (12, 13). However, the extent to which these genetic conditions independently increase the risk of iatrogenic coronary dissection during PCI remains unclear.

#### 3.1.3 Classification

**Figure 1:**
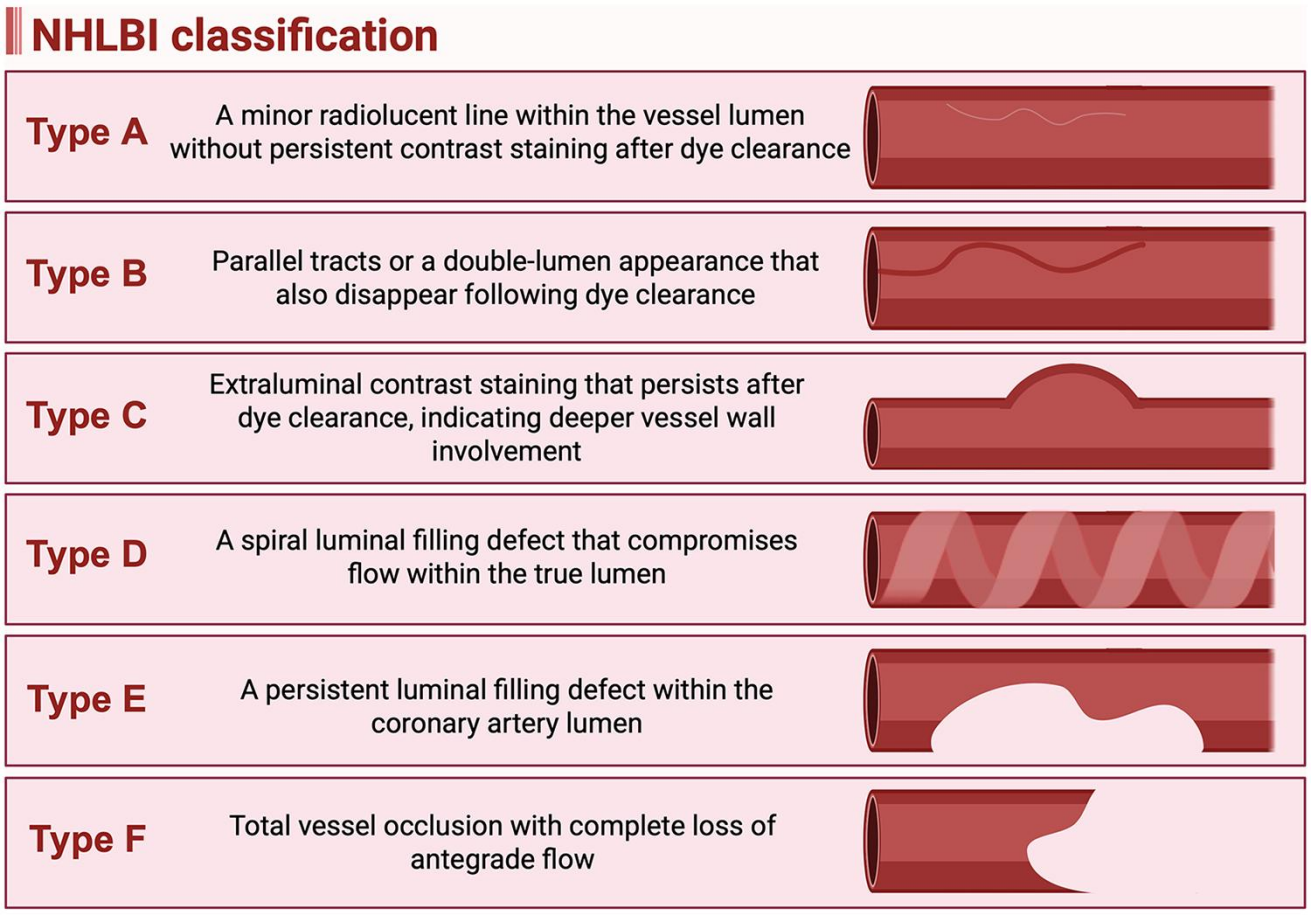
National heart, lung, and blood institute (NHLBI) classification of iatrogenic coronary artery dissection. (Created in BioRender. Attachaipanich, T. (2026) https://BioRender.com/mtbbyj5, licensed under Academic License).

Iatrogenic coronary dissection is classified by the National Heart, Lung, and Blood Institute (NHLBI) into six types (A-F) as shown in Figure 1 (14). Type A represents a minor radiolucent line within the vessel lumen without persistent contrast staining after dye clearance. Type B is characterized by parallel tracts or a double-lumen appearance that also disappear following dye clearance, similar to Type A. Type C involves extraluminal contrast staining that persists after dye clearance, indicating deeper vessel wall involvement. Type D is identified by a spiral luminal filling defect that compromises flow within the true lumen, while Type E presents as a persistent luminal filling defect within the coronary artery lumen. Type F represents the most severe form, defined by total vessel occlusion with complete loss of antegrade flow (14). The NHLBI classification correlates with both clinical outcomes and management strategies. Types C through F are typically clinically significant and often require intervention, whereas Types A and B are usually benign and may be managed conservatively (14). The risk of acute vessel closure increases with the severity of dissection, progressing from Type A to Type F (14). Types B and C are associated with relatively low incidences of acute vessel closure (3% and 10%, respectively), while Types D, E, and F carry substantially higher risks, with incidences of approximately 30%, 37%, and 69%, respectively (14). The specific management strategies for coronary dissection are discussed in the dedicated dissection section below.

#### 3.1.4 Management strategies

Management of guide catheter-induced dissection generally requires stent implantation, and the procedural approach depends on whether the guidewire has already crossed the lesion (10, 15). IVUS can help confirm whether the wire is positioned in the true or false lumen (10). If the wire is already across the lesion and located in the true lumen, a stent should be placed proximally to seal the entry point of the dissection and compress the hematoma (10). When the distal extent of the dissection is visualized, consider stenting the distal edge first to prevent further propagation (10, 15). If TIMI flow remains <3 after proximal stenting, this suggests persistent distal hematoma or occlusion; in such cases, balloon dilation or use of a cutting balloon may be considered to decompress the hematoma and restore flow (10, 15). If rewiring is necessary, IVUS should be used to ensure entry into the true lumen. IVUS is also useful for determining the length of the dissection, appropriate stent sizing, and optimization of stent deployment (10). Although optical coherence tomography (OCT) has been successfully used in some cases of catheter-induced dissection, its use should be approached with caution, as forceful contrast injection required for blood clearance can exacerbate the dissection (16).

If the wire has not crossed the lesion, an antegrade wiring attempt should be performed, followed by stenting once true lumen access is accessed (10, 15). When the initial wire lies within the false lumen, IVUS may be left in place alongside the first wire to provide real-time imaging guidance during the parallel wire technique. This approach allows the subintimal wire to tamponade the false lumen while a second wire is advanced into the true lumen (10). The subintimal transcatheter withdrawal (STRAW) technique may also be used to aspirate and decompress a subintimal hematoma that prevents distal reentry (17).

If antegrade wiring fails, alternative reentry techniques can be performed, including wire-based dissection reentry, reentry using a Stingray reentry system, or a retrograde approach (10, 15). The subintimal tracking and reentry (STAR) technique involves advancing a knuckled polymer-jacketed wire through the subintimal space until it spontaneously reenters the distal true lumen (18). Modified antegrade reentry methods, such as mini-STAR and limited antegrade subintimal tracking (LAST), have been developed to achieve earlier and more controlled reentry, minimizing the dissection plane and vessel trauma (18). The Stingray balloon system facilitates controlled subintimal navigation and targeted reentry into the true lumen using a flat, dual-lumen balloon and a specialized reentry wire (18). In selected cases with suitable collateral vessels, a retrograde approach may be performed.

#### 3.1.5 Preventive strategies

Prevention of iatrogenic coronary dissection includes avoiding contrast injection when a dampened pressure waveform is observed, preventing deep catheter engagement, maintaining coaxial guide alignment, minimizing aggressive wire manipulation, and exercising caution with polymer-jacketed guidewires (19). Preprocedural IVUS imaging can assist in lesion assessment and guide appropriate lesion preparation to minimize the risk of dissection (10).

### 3.2 Iatrogenic aortocoronary dissection

#### 3.2.1 Description

Aortocoronary dissection may extend into the aortic cusp or ascending aorta. The reported incidence of iatrogenic aortocoronary dissection ranges from 0.02% to 0.2%, with most cases originating from the right coronary cusp (7, 20). The majority of these dissections can be managed with ostial stenting, which seals the entry tear (20).

#### 3.2.2 Classification and management strategies

The Dunning classification provides an angiographic framework for characterizing aortocoronary dissections based on the extent of aortic involvement: Class I dissections are confined to the aortic cusp, Class II extend less than 4 cm into the ascending aorta, and Class III extend more than 4 cm into the ascending aorta (21). This classification correlates with both prognosis and management strategy. Higher Dunning classes are associated with an increased likelihood of surgical intervention and higher mortality, reported as 0% for Class I, 4.3% for Class II, and 6.5% for Class III (8). Most Class I cases can be successfully treated conservatively or with ostial stenting, whereas 17.4% of Class II and 40.3% of Class III cases require surgical repair (8). IVUS should be used to guide stent placement to ensure complete ostial coverage and seal the dissection entry point (22).

## 4 Intracoronary thrombus

### 4.1 Description and risk factors

Intracoronary thrombus is another common cause of acute vessel closure (6). It can result from inadequate antithrombotic therapy, a hypercoagulable state, or stent underexpansion (23). Several risk factors have been identified. Patient-related factors include male sex, smoking, hypercoagulable states, hyperglycemia, hypercholesterolemia, vasculitis, delayed presentation, and cardiogenic shock (23). Procedural- and lesion-related factors include acute plaque rupture, complex or long lesions, slow coronary flow, large vessel diameter (>4 mm), right coronary artery (RCA) involvement, old saphenous vein graft (SVG) lesions, inadequate anticoagulation, suboptimal dual antiplatelet therapy (DAPT), and heparin-induced thrombocytopenia (HIT) (23). Importantly, failure to confirm a therapeutic ACT before wiring the vessel is a preventable cause of intracoronary thrombus formation.

### 4.2 Management strategies

Optimal pharmacologic therapy plays a key role in reducing thrombus burden and improving procedural outcomes. DAPT, including aspirin and a potent P2Y_12_ inhibitor, along with intraprocedural anticoagulation, can reduce thrombus burden during PCI (24). In large randomized controlled trials, including ISAR-REACT 2 and EARLY ACS, routine pretreatment or intraprocedural administration of glycoprotein IIb/IIIa inhibitors (GPI) does not reduce ischemic events but is associated with an increased risk of bleeding complications (25–27). Consequently, current guidelines recommend against the routine upstream use of GPI; however, adjunctive intravenous or intracoronary GPI may be considered in the setting of a large thrombus burden or no-reflow to reduce infarct size and improve procedural success (24). In the INFUSE-AMI trial, a randomized controlled study of patients with large anterior STEMI, intracoronary abciximab was associated with a lower thrombus burden and smaller infarct size (28). Moreover, previous studies have shown that GPI administration is associated with a reduced thrombus burden in both ST-elevation MI (STEMI) and non-ST-elevation acute coronary syndrome (NSTE-ACS) (29, 30). Regarding the route of administration, randomized studies have demonstrated no significant difference between intravenous and intracoronary GPI in reducing thrombus burden (31, 32).

In addition to pharmacologic strategies, several mechanical techniques have been investigated for managing large thrombus burdens. Thrombus aspiration aims to remove intracoronary thrombus in this setting. In early randomized trials such as TAPAS study, thrombus aspiration was associated with improved TIMI flow and better clinical outcomes (33, 34). However, subsequent large randomized controlled trials, including TASTE and TOTAL trials, found that routine thrombus aspiration did not improve clinical outcomes and was associated with an increased risk of stroke (35, 36). A pooled analysis of three large randomized studies similarly showed no clinical benefit with routine thrombus aspiration (37). In contrast, subgroup analyses suggested that in patients with a high thrombus burden, thrombus aspiration was associated with a significantly lower risk of cardiovascular death, though it was offset by an increased risk of stroke (37). Overall, current guidelines recommend against the routine use of thrombus aspiration in STEMI; however, it may be considered in cases of a large thrombus burden, risk of no-reflow, or as a bailout strategy following failed balloon angioplasty or stent deployment (24). A recent prospective study reported that mechanical thrombus aspiration in acute coronary syndrome (ACS) with a high thrombus burden achieved a high rate of thrombus removal and improved myocardial perfusion (38). Nonetheless, randomized data supporting mechanical thrombus aspiration remains limited.

Intracoronary fibrinolytic therapy is another potential treatment option for large thrombus burden. Several small randomized trials have demonstrated the potential efficacy and safety of intracoronary fibrinolytic agents in STEMI populations (39–41). In the DISSOLUTION trial, a small randomized study of STEMI patients with a high thrombus burden showed that intracoronary urokinase improved myocardial blood flow without increasing bleeding risk (42). However, current evidence remains limited to small randomized studies, and further large-scale trials are needed.

### 4.3 Preventive strategies

Because procedural thrombus formation arises from mechanical disruption, inadequate anticoagulation, or suboptimal stent deployment, the primary focus in PCI-related thrombosis should be on optimized intraprocedural anticoagulation, careful lesion preparation, and proper stent expansion, with selective use of adjunctive therapies in cases where thrombus develops despite these preventive measures. Overall, evidence from STEMI studies may guide the treatment of iatrogenic intracoronary thrombus, but its occurrence during PCI should be recognized as a distinct procedural complication requiring prompt identification and management.

## 5 No-reflow phenomenon

### 5.1 Description and risk factors

The no-reflow phenomenon refers to impaired myocardial reperfusion despite successful epicardial coronary revascularization. Although advances in intervention techniques have reduced its incidence, no-reflow remains associated with adverse outcomes, including heart failure, arrhythmia, cardiogenic shock, and mortality (43). The no-reflow phenomenon is more likely to occur in the setting of STEMI, degenerated SVG, and during rotational atherectomy (44, 45). Predictors included patient-related factors such as advanced age, male sex, smoking, dyslipidemia, chronic kidney disease (CKD), diabetes mellitus, hypertension, CHA_2_DS_2_-VASc ≥ 5, elevated inflammatory and cardiac biomarkers, and higher Killip class (44, 46–48). Procedural- and lesion-related factors include lesion length >15 mm, prolonged reperfusion time, SVG PCI, low pre-procedural TIMI flow grade, high thrombus burden, and intra-aortic balloon pump (IABP) use, (49–52). Among patients with STEMI, the presence of atrial fibrillation, late presentation, and prolonged door-to-balloon time also increase the risk of no-reflow (45, 53).

The mechanisms underlying the no-reflow phenomenon include distal embolization, ischemic injury, and reperfusion injury (44). Distal embolization is the most common cause, resulting from platelet activation, thrombus fragmentation, and the release of plaque debris during catheter manipulation (44). Ischemic injury leads to cellular swelling, loss of vascular tone, and interstitial edema, which together cause microvascular obstruction and impaired perfusion (44). Reperfusion injury involves inflammatory cell infiltration, cytokine release, and oxidative stress, resulting in endothelial dysfunction and tissue injury (44).

The diagnosis of no-reflow is made angiographically, defined as a TIMI flow grade <3 and a myocardial blush grade (MBG) < 3 in the absence of other causes. These findings indicate abnormal myocardial perfusion despite epicardial coronary artery revascularization (44, 45). Other potential causes of impaired flow, such as macrovascular dissection, vasospasm, or thrombus, should be excluded using intravascular imaging modalities such as intravascular ultrasound (IVUS) (54).

### 5.2 Management strategies

The management of coronary no-reflow includes the administration of vasodilators, assessment of anticoagulation adequacy through activated clotting time (ACT) monitoring, and hemodynamic stabilization with circulatory support as needed (55). Intracoronary administration of vasodilators, delivered via a microcatheter, dual lumen catheter, or an over-the-wire balloon, is preferred to minimize systemic side effects and ensure targeted delivery to the microcirculation. Commonly used vasodilators include adenosine, sodium nitroprusside, and calcium channel blockers (CCBs) such as verapamil, diltiazem, and nicardipine, as well as intracoronary epinephrine as shown in Table 1 (56).

**Table 1:** Pharmacotherapy vasodilators in the management of coronary no-reflow.

Vasodilator	Route	Dose
Adenosine	Intracoronary	50–200 μg
	Intravenous	70 μg/kg/min
Diltiazem	Intracoronary	400 μg
Epinephrine	Intracoronary	50–200 μg
Nicardipine	Intracoronary	50–200 μg
Nitroprusside	Intracoronary	50–200 μg
Verapamil	Intracoronary	100–250 μg

While adenosine and nitroprusside have been shown to improve angiographic TIMI flow, data demonstrating a corresponding reduction in major adverse cardiac events (MACE) are limited. In the AMISTAD and AMISTAD-II randomized controlled trials, adenosine administration was associated with a significant reduction in infarct size; however, no improvement in clinical outcomes was observed (57, 58). A randomized study in STEMI patients following thrombus aspiration compared adenosine, nitroprusside, and saline, and demonstrated that only adenosine was associated with greater ST-segment resolution (59). Angiographic microvascular obstruction and 30-day MACE were numerically lower with adenosine but did not reach statistical significance, while no difference was observed in the nitroprusside group (59). A subsequent randomized study using higher doses of adenosine reported no significant improvement in infarct size or microvascular obstruction, but did show an increased incidence of adverse events, including heart failure. Notably, the adenosine dose used in this trial (1–2 mg) was substantially higher than the recommended intracoronary dose of 50–200 μg (60, 61). A meta-analysis of nitroprusside in STEMI patients demonstrated a significant reduction in the incidence of no-reflow with nitroprusside therapy (62).

Current evidence for CCBs in the management of no-reflow remains limited. Only small randomized studies have evaluated the efficacy of CCBs in this setting (63–65). Meta-analyses demonstrated that intracoronary verapamil and diltiazem were associated with a reduced incidence of no-reflow and lower rates of MACE at 6 months, although without improvement in left ventricular ejection fraction (66, 67). A network meta-analysis comparing various intracoronary agents in STEMI showed that verapamil, nitroprusside, and adenosine were associated with lower rates of no-reflow compared with control (68). Further large-scale randomized trials are needed to clarify the benefits of CCBs in this context.

Intracoronary epinephrine has also been investigated as a treatment option for no-reflow that results in coronary microvascular vasodilation through β_2_-adrenergic receptor activation, which promotes smooth-muscle relaxation and reduces intracellular calcium overload. A small retrospective study demonstrated that intracoronary epinephrine improved coronary blood flow in STEMI patients with no-reflow refractory to conventional therapy (69). Similarly, a small randomized trial comparing intracoronary epinephrine with adenosine found that epinephrine achieved a significantly higher frequency of TIMI grade 3 flow and a lower corrected TIMI frame count compared with adenosine (70). An important advantage of epinephrine over traditional coronary vasodilators is its ability to augment systemic blood pressure, which is particularly beneficial in no-reflow accompanied by hypotension or cardiogenic shock, where other vasodilators may worsen hemodynamics.

### 5.3 Preventive strategies

Preventive strategies for no-reflow should address both precatheter (71) ization and intraprocedural factors based on patient risk profiles (56). Precatheterization measures include optimization of comorbidities and strict glycemic control, while minimizing door-to-balloon time remains essential. Intraprocedural prevention includes the prophylactic use of intracoronary vasodilators and selective thrombus aspiration in cases of high thrombus burden (56). Use of embolic protection devices may also be considered in setting of SVG PCI, since SVG PCI are associated with an increased risk of no-reflow compared with native vessel PCI (71). Early randomized studies in SVG PCI demonstrated that distal embolic protection was associated with a significantly lower incidence of no-reflow (72). Similarly, a randomized study in ACS patients with attenuated plaque ≥5 mm in length identified by IVUS found that distal embolic protection was associated with a lower risk of no-reflow compared with control (73). Therefore, current guidelines recommend considering embolic protection devices for SVG PCI as a Class IIa indication (71, 74).

## 6 Coronary artery perforation

### 6.1 Description

Coronary artery perforation is an uncommon but potentially life-threatening complication of PCI, with a reported incidence ranging from 0.2% to 1.5% of all PCI procedures (75–77). In chronic total occlusion (CTO) interventions, the incidence is substantially higher, reaching up to 4.8% (78). Coronary perforation is associated with an increased risk of MACE and in-hospital mortality of up to 17.8% in some studies, as well as with increased long-term mortality (75–77).

### 6.2 Risk factors

Predictors include patient-related factors such as advanced age, female sex, CKD, hypertension, current smoking, prior MI, previous coronary artery bypass grafting (CABG), lower body mass index, presentation with cardiogenic shock, and prior PCI (75–77, 79). Anatomical factors include CTO lesions, high-risk type C morphology, bifurcation lesions, heavy calcification, long lesion length, small vessel diameter, and severe vessel tortuosity (75–77, 79–81). Procedural factors include the use of hydrophilic-coated, polymer-jacketed or stiff-tip guidewires, oversized balloons and stents, left main or left anterior descending (LAD) artery intervention, use of laser or rotational atherectomy, preprocedural TIMI flow <2, and preexisting coronary dissection (75–77, 79–81). In setting of CTO interventions, predictors of coronary perforation include advanced age, use of anterograde dissection and re-entry or retrograde approaches, presence of blunt or no stump morphology, and moderate-to-severe calcification (82). Importantly, the use of intracoronary imaging-guided PCI has been shown to significantly reduce the risk of coronary perforation, particularly in the context of CTO or heavy calcification (83).

Coronary artery perforation can be classified based on the severity of the lesion and the size of the affected vessel. Angiographic classification systems describe the severity according to the extent of extravasation, which correlates with the underlying etiology, clinical impact, and prognosis. Classification based on the vessel size also guides management strategies.

### 6.3 Classification

**Figure 2:**
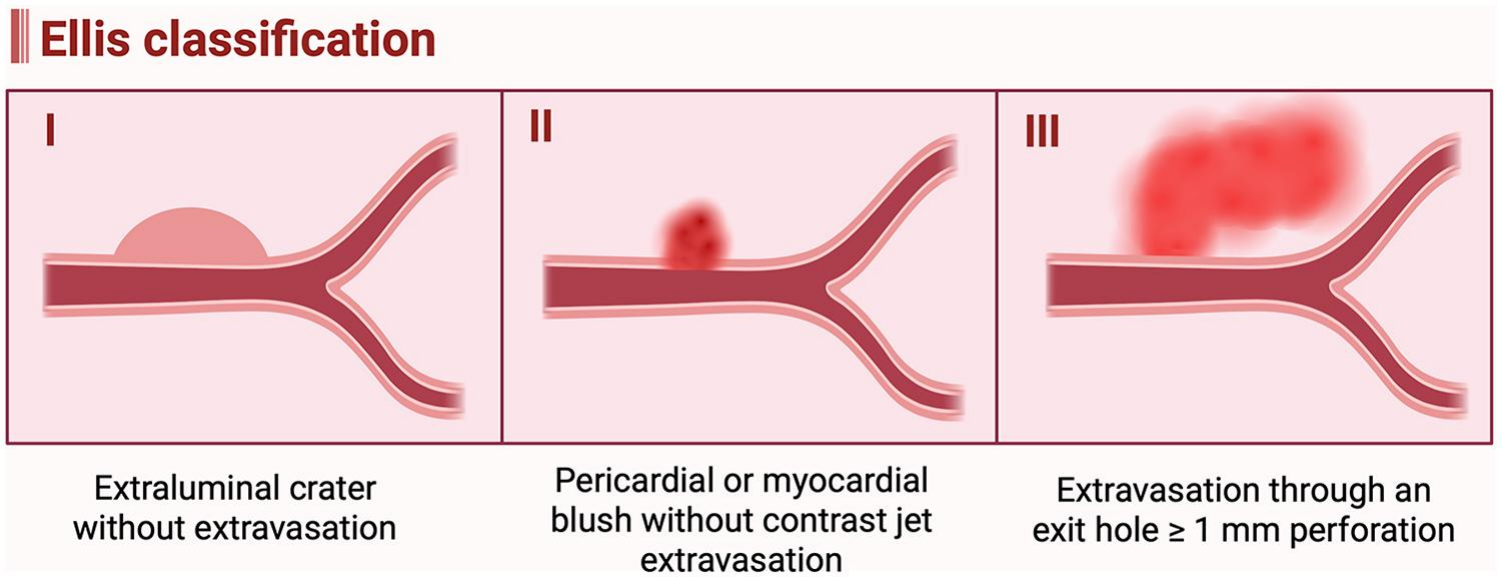
Ellis classification of coronary artery perforation. (Created in BioRender. Attachaipanich, T. (2026) https://BioRender.com/rbq4kvi, licensed under Academic License).

The Ellis classification was proposed to categorize coronary artery perforation based on the angiographic severity of contrast extravasation and remains the most widely used grading system as shown in Figure 2 and Table 2 (84). Type I refers to a concealed perforation, characterized by an extraluminal crater without any evidence of contrast extravasation. Type II denotes a limited perforation, in which localized or perivascular contrast staining is present but without a visible jet of contrast extravasation. Type III represents a severe perforation, defined by a free-flowing stream of contrast extravasation through an exit hole ≥1 mm in diameter, with contrast filling and clearing outside the vessel lumen (84). In Type III perforations, contrast may extravasate into the pericardial space, potentially leading to cardiac tamponade, or into a cardiac chamber, which generally results in better hemodynamic tolerance. When extravasation extends into an anatomical cardiac chamber, the perforation is subclassified as Type III cavity spilling.

**Table 2:** Ellis classification of coronary artery perforation: definitions, clinical outcomes, and recommended management strategies.

Ellis type	I	II	III	III CS
Definition	Extraluminal crater without extravasation	Pericardial or myocardial blush without contrast jet extravasation	Extravasation through an exit hole ≥1 mm perforation	Perforation into an anatomical cavity chamber
Clinical outcome	Usually benign, hemodynamic stable	Usually benign, hemodynamic stable, risk of delay tamponade	Hemodynamic compromise, high risk of tamponade and mortality	Can be benign, better hemodynamic tolerance
Management	– Observation if clinical stable – Hemodynamic support – Prolong balloon inflation – Consider anticoagulation reversal	– Prolong balloon inflation – Hemodynamic support – Pericardiocentesis if cardiac tamponade – Consider covered stent – Consider coil embolization if distal vessel perforation – Consider anticoagulation reversal	– Prolong balloon inflation – Hemodynamic support – Pericardiocentesis if cardiac tamponade – Covered stent – Consider anticoagulation reversal	– Prolong balloon inflation – Consider covered stent in hemodynamic unstable – Consider anticoagulation reversal

The Ellis classification correlates with both the mechanism of perforation and clinical outcomes. Types I and II typically result from small-vessel or distal guidewire perforations and are usually hemodynamically stable. In contrast, Type III perforations are often associated with large-vessel injuries, hemodynamic compromise, and a significantly higher risk of major complications and mortality (85). A retrospective study reported in-hospital mortality rates of 0%, 1.7%, and 21% for Ellis Types I, II, and III, respectively. During long-term follow-up, Ellis Type III perforations were associated with a threefold increase in all-cause mortality compared with Types I and II (85). The incidence of acute cardiac tamponade was also significantly higher in Type III perforations (40.4%) compared with Types I and II (3.8%), whereas the rate of delayed tamponade was higher in Types I and II (7.7%) than in Type III (3.5%) (85).

### 6.4 Large-vessel perforation

#### 6.4.1 Description

Large-vessel coronary perforation most commonly occurs due to the use of oversized or overinflated balloons and stents, or as a complication of atherectomy devices such as rotational atherectomy (86). These perforations are typically classified as Ellis Type III, representing the most severe form of coronary perforation (85, 86). Clinically, large-vessel perforation can lead to rapid hemodynamic deterioration due to cardiac tamponade, often requiring emergent pericardiocentesis and hemodynamic support (86). The sudden onset of acute chest pain or hemodynamic instability during balloon inflation or stent deployment should raise suspicion for coronary artery perforation, which can be promptly confirmed by angiographic visualization of contrast extravasation.

#### 6.4.2 Management strategies

Management of coronary artery perforation involves both general supportive measures applicable to all cases and specific strategies based on the vessel size and perforation severity. General management includes hemodynamic stabilization with fluid resuscitation, vasopressor support if needed, and pericardiocentesis in cases of cardiac tamponade (81). Importantly, in cases of ongoing extravasation or persistent localized tamponade, particularly in patients with prior CABG, where the pericardial space may not be amenable to complete percutaneous drainage, emergent surgical intervention, including creation of a pericardial window or operative repair, may be required to restore and maintain hemodynamic stability (87).

Balloon tamponade remains the first-line procedural intervention and should be performed by inflating a balloon at or just proximal to the perforation site for 5–10 min using a balloon-to-artery ratio of 1:1 at low inflation pressure (81). Because prolonged balloon inflation can compromise distal myocardial perfusion and lead to ischemia or infarction, a specialized device known as the Ringer perfusion balloon has been developed. This helical-shaped inflatable balloon opposes the vessel wall while maintaining antegrade coronary perfusion through a hollow central lumen (88). In a recent study, the Ringer perfusion balloon demonstrated a high procedural success rate, achieving cessation of contrast extravasation in 84.6% of cases and maintenance of TIMI grade 2–3 flow in 100% of patients (88). Intravenous anticoagulant and antiplatelet therapies should be withheld immediately following the recognition of a coronary artery perforation. Reversal of anticoagulation may be considered once all interventional equipment has been withdrawn from the coronary artery. Recent evidence further supports the safety of this approach (89). In a single-center retrospective study 160 patients with coronary perforations during CTO PCI, protamine administration after complete device removal was associated with significantly lower rates of in-hospital mortality and cardiac tamponade, with no observed cases of acute stent thrombosis (89). These findings reinforce that appropriately timed heparin reversal can be safely utilized in select cases of coronary artery perforation.

If balloon tamponade fails to control the bleeding, covered stent implantation should be performed (15). The goal of a covered stent is to seal the perforation using an impermeable barrier layer, thereby restoring vessel integrity and preventing further extravasation (90, 91). Two main types of covered stents are currently available. The Graftmaster (Abbott Vascular) has a traditional “sandwich” design, consisting of a bilayer metallic framework with a thin, biocompatible polytetrafluoroethylene (PTFE) membrane between the layers. It typically has a larger diameter and requires a larger guide catheter for delivery (91). The PK Papyrus (Biotronik) has a single-layer cobalt-chromium platform covered with a highly elastic polyurethane membrane, offering a smaller profile, improved flexibility, and compatibility with smaller guide catheters and standard 0.014-inch guidewires (90, 91).

The deployment technique depends on the guide catheter size and stent type. If the guide catheter is large enough, the “block-and-deliver” technique can be used with a single guide catheter (92). In this method, a second guidewire is advanced while the balloon remains inflated at the perforation site. The balloon is briefly deflated to allow guidewire passage, then reinflated to maintain hemostasis while the covered stent is advanced proximally. Once positioned, the balloon is deflated and withdrawn, allowing deployment of the covered stent at the perforation site (92).

When the initial guide is too small, the “ping-pong” (dual-guide) technique can be performed (93). In this approach, a second guide catheter is introduced while maintaining balloon inflation. The balloon is briefly deflated to allow passage of the second guidewire and covered stent, to the perforation site. After the stent is positioned, the first guidewire and balloon are withdrawn, and the covered stent is deployed to seal the perforation (93).

Despite their efficacy, covered stents cause side-branch occlusion, which may result in myocardial ischemia. Evidence comparing different covered stent types remains limited. Observational study in patients with large-vessel perforations successfully sealed with a covered stent reported that the PK Papyrus was associated with a significantly lower rate of target lesion revascularization at 30 days compared with the Graftmaster, although no differences were observed in 30-day or 1-year MACE (94).

Although covered stents provide rapid and effective sealing of large-vessel perforations, several concerns remain regarding their long-term performance, including higher rates of restenosis and stent thrombosis (91). While these devices were conceptually designed to reduce in-stent restenosis by creating a mechanical barrier that limits neointimal proliferation through the stent struts, randomized data in SVG-PCI have not demonstrated a reduction in restenosis compared with bare-metal stents (95, 96). A retrospective study reported a definite stent thrombosis rate of 8.6% among patients with Ellis Grade III perforation treated with a covered stent, and registry data similarly show a higher incidence of stent thrombosis within one year in covered stents compared with other stent platforms (96, 97). These findings underscore the need for optimal deployment technique and high-pressure post-dilation.

Perforations occurring in or near coronary bifurcations represent a particularly challenging scenario, as management must balance rapid hemostasis with preservation of side-branch flow. Initial treatment follows standard principles, including immediate balloon tamponade and hemodynamic stabilization, with precise localization of the perforation relative to the bifurcation determined using angiography and intravascular imaging. When bleeding persists, covered stent implantation is often required; however, bifurcation perforations may necessitate intentional side-branch jailing to achieve complete sealing (98). A systematic review of Ellis grade III perforations involving left main distal bifurcation lesions demonstrated that crossover covered stenting is the most commonly employed strategy, while alternative approaches, including covered stent implantation with the jailed balloon technique or simultaneous kissing covered stents, may improve side-branch preservation in selected cases (98). Side-branch sacrifice is sometimes unavoidable to achieve definitive hemostasis, with subsequent reaccess using high tip-load guidewires and dual-lumen catheters when clinically indicated. These principles are illustrated by a recent case report describing a complex bifurcation core perforation managed with a dual-covered stent strategy, in which intentional side-branch jailing was required for sealing, followed by advanced rewiring techniques to restore coronary blood flow (99).

### 6.5 Distal-vessel perforation

#### 6.5.1 Description

Distal-vessel perforation typically occurs in distal coronary segments, side branches or collaterals in patients undergoing CTO PCI, most commonly resulting from guidewire-related injury. Previous studies have shown that the majority of guidewire-associated perforations are caused by hydrophilic guidewires (100). Compared with large-vessel perforation, small-vessel perforation carries a lower risk of immediate hemodynamic compromise, but it may lead to delayed cardiac tamponade, which can develop hours to days after PCI (101). Diagnosis is usually established by coronary angiography, which should include prolonged image acquisition with panning of the distal vessel to visualize subtle or delayed contrast extravasation. Adjunctive imaging with transthoracic echocardiography, including contrast-enhanced echocardiography, can determine the presence and location of ongoing extravasation, differentiate pericardial from intracardiac leakage, and guide the need for additional interventions such as coil embolization or covered stent placement.

#### 6.5.2 Management strategies

Management principles are similar to those used in large-vessel perforation and begin with prolonged balloon inflation (15). If balloon tamponade fails to achieve hemostasis, a microcatheter should be advanced as close as possible to the perforation site for embolization therapy (15). Coil embolization is the preferred therapeutic option for sealing distal or small-vessel perforations vessel (81, 102, 103). Based on their delivery mechanism, coils are categorized into two main types. Pushable coils are non-retrievable once deployed and have a less predictable final position, whereas detachable coils permit controlled, reversible delivery, allowing for more precise placement at the target site (81, 103). During coil delivery, a second guidewire and balloon can be inflated proximally to the perforated branch to control bleeding, using the block-and-deliver technique (92).

In case where coils are unavailable, several alternative embolic agents may be used to achieve hemostasis. These include autologous fat, typically harvested from the abdomen or groin and delivered through a microcatheter; autologous blood clot, which serves as a natural occlusive material; and thrombin, which promotes localized thrombosis at the site of perforation to effectively seal the vessel (81). A negative suction technique using a microcatheter connected to a syringe has also been reported to achieve successful hemostasis by collapsing the perforated distal vessel (104). More recently, a novel technique involving the delivery of silk or absorbable suture fragments via microcatheter has been reported, effectively occluding distal perforations and achieving durable hemostasis (105). If embolization fails to seal the perforated branch, a covered stent can be deployed in the main vessel to seal the ostium of the perforated side branch, thereby preventing further extravasation (15).

### 6.6 Reversal of anticoagulation

Reversal of anticoagulation can facilitate hemostasis in cases of coronary artery perforation, but it must be balanced against the increased risk of thrombotic complications, particularly when interventional devices remain within the coronary artery. Reversal is generally recommended only after all equipment has been removed from the coronary system (15). Several studies have reported that a notable proportion of patients undergo anticoagulation reversal while devices are still positioned within the coronary artery (75, 106). However, evidence regarding the safety of this practice remains limited. A recent retrospective study demonstrated that although heparin reversal during active instrumentation was associated with successful hemostasis, it also resulted in coronary thrombosis in 7.6% of cases (107). Furthermore, a minimum ACT <150 s was strongly correlated with the occurrence of coronary thrombosis (107). These findings highlight the importance of careful timing of anticoagulant reversal.

More recently, additional evidence has demonstrated that protamine administration can be safely performed once all intracoronary equipment has been removed. In a single-center retrospective study of 160 patients who developed coronary perforation during CTO PCI, protamine administered after complete device withdrawal was associated with substantially lower rates of in-hospital mortality and cardiac tamponade, with no cases of acute stent thrombosis observed (89). These data support the safety of anticoagulation reversal when undertaken at the appropriate procedural stage.

For reversal, unfractionated heparin can be partially or fully neutralized with protamine sulfate, administered at a dose of 1 mg intravenously for every 100 units of heparin given, with a typical total dose of 25–50 mg (15, 108). In cases where bivalirudin is used, although it has a short plasma half-life, fresh frozen plasma may be considered to partially reverse its effect and promote more rapid hemostasis (108).

## 7 Entrapped equipment

Entrapped equipment is rare and can result in abrupt vessel closure, MI, coronary perforation, thrombosis, or arrhythmia (109). In severe cases, device entrapment may necessitate emergency surgical retrieval (109). The reported incidence of device entrapment ranged between 0.05% and 0.4% (109). Risk factors for device entrapment include severe vessel angulation, heavy calcification, and marked vessel tortuosity (109). The use of intravascular imaging (IVUS or OCT) to assess lesion characteristics before intervention can help guide appropriate lesion preparation and potentially reduce the risk of device entrapment. However, these imaging catheters themselves can become entrapped in tight or heavily calcified lesions, and therefore, forceful advancement should be avoided (110).

### 7.1 Coronary guidewires entrapment

#### 7.1.1 Description

The incidence of guidewire entrapment or fracture ranges from 0.1%–0.2%, increasing to approximately 0.5% in CTO interventions (109, 111, 112). Risk factors for guidewire entrapment and fracture include guidewire jailing, excessive rotation (>180°), aggressive wire manipulation, use of multiple guidewires, and advancing an atherectomy burr over a kinked wire (113, 114). Anatomical features include marked vessel angulation, heavy calcification, bifurcation lesions, and CTO morphology further increase the risk of entrapment (113, 114). Entrapment may lead to wire fracture and retained fragments, particularly when excessive traction is applied. Advancing a rotational atherectomy burr against kink or immobile wire can also cause transection (113, 114).

Diagnosis of guidewire fracture is typically made by identifying loss of wire continuity on fluoroscopy or confirmed using IVUS (115). Retained guidewire fragments can result in serious complications, including thrombosis, coronary dissection, perforation, arrhythmia, or systemic embolization (116). Structurally, guidewires consist of inner and outer components; unraveling of the outer coil increases thrombogenicity and may cause luminal obstruction or distal embolism (109).

#### 7.1.2 Management strategies

Management of an entrapped guidewire typically begins with percutaneous retrieval techniques. A microcatheter can be advanced as distally as possible over the trapped wire, followed by gradual withdrawal to facilitate release (15, 117). Alternatively, a small-profile balloon may be advanced, inflated at the site of entrapment, and gently pulled back to dislodge the trapped segment (117). Excessive traction should be avoided, as the guidewire core may unravel proximally and extend into the aorta. This unravelling can be difficult to visualize angiographically and is highly thrombogenic, emphasizing the importance of controlled retrieval rather than forceful pulling. If percutaneous retrieval fails, intentional wire transection can be performed. This technique has been described using rotational atherectomy to cut the entrapped guidewire, retrieve the proximal broken end, and leave the remaining distal fragment securely jailed beneath a stent, thereby reducing the risk of embolization (118). Surgical removal considered if the fragment poses thrombotic or embolic risk (117).

In setting of retained guidewire fragments, management should be individualized based on the location and extent of the retained wire, given its thrombogenic potential and the limited evidence regarding long-term outcomes. Conservative management may be appropriate in selected cases, particularly when the fragment is confined to a small, distal, or chronically occluded vessel, or when the risks of percutaneous or surgical retrieval outweigh potential benefits (111). Several studies have reported a benign clinical course in patients with retained fragments confined to chronically occluded or distal vessels, or in cases where retrieval attempts were unsuccessful (111, 119).

However, if the wire fragment extends into the aorta, removal is recommended due to the risk of thrombus formation and systemic embolization (111). Percutaneous retrieval techniques include the snare technique and the double- or triple-wire twisting method, in which additional guidewires are advanced alongside the fractured segment and rotated using a torquer to entangle and retrieve the wire (111, 120, 121). The knuckle-twister technique, a modification using a polymer-jacketed tapered wire formed into a knuckle, can be advanced distal to the fracture, then rotated and withdrawn to capture and extract the fragment, with high reported success and reproducibility (122). If percutaneous retrieval fails, surgical removal should be considered (111, 120).

For fragments confined within the coronary artery, several percutaneous strategies are available, including the snare technique, wire-twisting technique, small-balloon inflation and withdrawal, or stent deployment to seal and exclude the fractured wire from the lumen (111, 117, 120). When these percutaneous approaches are unsuccessful or carry excessive procedural risk, surgical removal should be pursued, whereas conservative management may be reserved for high-risk or clinically stable cases (111, 117).

#### 7.1.3 Preventive strategies

Coronary guidewire entrapment can be reduced by careful lesion assessment, appropriate wire selection, and avoidance of excessive wire rotation (>180°), particularly with hydrophilic or polymer-jacketed guidewires. Resistance during manipulation should prompt reassessment rather than forceful advancement, guidewire jailing should be minimized, and rotational atherectomy should not be performed over kinked or immobile guidewires, with frequent fluoroscopic confirmation of wire position in complex anatomy.

### 7.2 Rota burr entrapment

#### 7.2.1 Description

Previous studies have reported an incidence of approximately 0.4%–1% of procedures (123). The rotational burr, coated with diamond particles, ablates plaque in a unidirectional manner. When burring is halted within a tight or calcified segment, particularly distal to the lesion, the burr may become irretrievably trapped (124). One common mechanism is the “Kokesi phenomenon”, named after the Japanese doll with a bulb-shaped head and hollow body (124). This occurs when a small burr is advanced at high rotational speed through a narrow, rigidly calcified lesion, mimicking the insertion of the doll's neck into its body, allowing rotation but preventing withdrawal (124). Conversely, using an oversized burr in a severely calcified vessel or maintaining a high burr-to-artery ratio can also increase the risk of entrapment (125). Additionally, performing rotational atherectomy on a recently implanted, underexpanded stent may predispose to burr entrapment, as the stent struts impede burr movement (125).

#### 7.2.2 Management strategies

When burr entrapment occurs, percutaneous retrieval should be attempted prior to emergency surgery. The initial management involves balloon dilation of the lesion to enlarge the lumen and release the burr. This can be achieved by introducing a second guidewire, either via a second guiding catheter or by removing the rotablation advancer sheath to allow wire passage (125, 126). After successful wiring, balloon angioplasty at or proximal to the burr may facilitate its retrieval (125). Additional techniques include pulling the RotaWire, burr, and guiding catheter as a single unit, using the distal wire bulb to transmit traction and dislodge the burr, as well as disconnecting the burr from the housing and rotating it in reverse while maintaining gentle traction to free it from the lesion. If unsuccessful, deep guide catheter intubation or the use of a guide-extension (“mother-and-child”) system, advancing a guide-extension or smaller guide catheter toward the burr, may assist in extraction (125, 127, 128). The snare technique has also been reported as an effective percutaneous retrieval method (129). If all percutaneous attempts fail, emergent surgical removal should be performed (125, 130).

#### 7.2.3 Preventive strategies

Preventive strategies include avoiding excessive forward force, refraining from stopping burr rotation within or distal to the lesion, and preventing abrupt deceleration of rotational speed (125). Appropriate burr sizing and gradual advancement using a pecking motion remain essential procedural principles to minimize the risk of entrapment (125).

### 7.3 Balloon rupture and entrapment

#### 7.3.1 Description

Balloon entrapment is an uncommon but can lead to abrupt vessel closure and myocardial ischemia. It may occur secondary to balloon shaft fracture, balloon rupture, failure to deflate, or interaction with a previously implanted stent struts (131). The mechanisms underlying failure to deflate include elastic recoil of a severely calcified lesion compressing the balloon, premature withdrawal into the guide catheter before complete deflation, or kinking or damage to the hypotube (132).

#### 7.3.2 Management strategies

Management of failure to deflate includes using an indeflator filled with normal saline rather than contrast, attempting gentle balloon withdrawal without full deflation, or puncturing the balloon with another guidewire (133). Increasing the proportion of saline in the contrast mixture reduces viscosity and shortens balloon deflation time (132). If these steps are unsuccessful, high-pressure inflation to intentionally rupture the balloon may be used; however, this approach should be reserved for large-caliber vessels due to the high risk of coronary dissection or perforation (132).

When balloon entrapment occurs, and the cause is failure to deflate, the above steps should be followed. Otherwise, strategies include repeated gentle inflation and deflation, or gently forward-backward manipulation to release the trapped balloon (15, 131). Several techniques can be performed for retrieval of an entrapped balloon. The balloon-trapping technique involves advancing a second balloon into the guide catheter and inflating it to secure the entrapped balloon shaft, allowing the entire system to be withdrawn as a single unit. Alternatively, advancement of a guide-extension catheter over the trapped balloon shaft can facilitate its release by providing additional support and coaxial alignment. The snare technique may also be used when the balloon is accessible (15, 131). In cases where entrapment is caused by a heavily calcified lesion, plaque modification using a second guidewire and balloon to dilate the vessel distal to the trapped segment can modify the and release the entrapped balloon (134). The mini-STAR technique has also been described as an effective bailout option (135).

Balloon shaft fracture may occur as a result of kinking or excessive manipulation. Management depends on the location of the retained fragment (131). If a portion of the fragment remains within the guide catheter, a balloon-trapping maneuver or snare device may be used for retrieval (131). Surgical removal should be considered if percutaneous efforts are unsuccessful. When the fragment extends beyond the guide catheter, retrieval can be attempted by inflating another balloon alongside the retained segment to facilitate extraction or by using a snare system (131). Rotational atherectomy has also been successfully used to modify calcified plaque surrounding a retained balloon fragment, followed by stent implantation to secure the material and prevent distal embolization (136).

#### 7.3.3 Preventive strategies

Preventive strategies include adequate lesion preparation, particularly with atherectomy for severely calcified lesions, avoiding very high-pressure balloon inflation, and avoiding forceful advancement of a balloon through resistant or underprepared segments.

### 7.4 Microcatheters entrapment

#### 7.4.1 Description

The mechanisms include over-torquing, forceful advancement through heavily calcified or tortuous vessels, and use of the microcatheter without an internal guidewire for support (131). These factors increase friction and mechanical stress along the shaft, predisposing to device entrapment or structural failure.

#### 7.4.2 Management strategies

The management approach is generally similar to that used for other types of device entrapment (15, 131). Management options include percutaneous retrieval, surgical removal, or conservative management, depending on the fragment's location and clinical impact. Among percutaneous strategies, the trapping technique is often effective (15, 131, 137). Additional options include the snare technique or plaque-modification maneuvers, such as advancing a second guidewire with balloon angioplasty to relieve mechanical impingement and facilitate removal (15, 131). If percutaneous retrieval fails, surgical extraction may be required, though conservative management can be reasonable when a small fragment remains confined to a distal branch and the procedural risk outweighs the benefit (15, 131).

#### 7.4.3 Preventive strategies

Preventive strategies focus on minimizing mechanical stress during catheter manipulation. Operators should avoid excessive rotation and refrain from aggressive advancement through resistant or heavily calcified segments. Selecting microcatheters with reinforced or stiffer distal tips may further reduce the risk of tip fracture or deformation (138).

## 8 Stent loss

Stent loss is a rare complication of PCI that can lead to systemic embolization, emergency CABG, or even death (139). The incidence of stent loss has declined with the introduction of premounted stents and improvements in device design, but it remains clinically relevant, with reported rates ranging from 0.3% to 1.3% (139, 140). Lesion-related risk factors include severe calcification and pronounced proximal angulation (139). Procedural contributors include inadequate vessel preparation or direct stenting without predilation, forceful advancement or withdrawal, use of guide-extension catheters, and stent delivery through a previously implanted stent (139, 140). Based on these risk factors, stent loss can be minimized by ensuring thorough lesion preparation, avoiding forceful advancement when resistance is encountered, using guide-extension catheters to facilitate controlled stent delivery across calcified or tortuous segments, and delaying negative balloon preparation until the stent has reached the intended deployment site.

Stent types used in contemporary PCI include bare-metal stents (BMS), drug-eluting stents (DES), and bioresorbable scaffolds (BRS). BRS are designed to provide temporary mechanical support followed by complete resorption, with the theoretical advantage of avoiding long-term complications associated with permanent metallic stents, such as in-stent restenosis and late stent thrombosis (141). Compared with metallic stents, BRS have thicker struts and reduced radial strength, which may increase susceptibility to deformation during delivery and predispose to complications such as scaffold unloading or detachment. Case reports have described successful management of BRS unloading with immediate *in-situ* balloon expansion and post-dilation, although the relative risk of stent loss across different stent platforms remains unclear (142, 143).

### 8.1 Partial stent loss

Partial stent loss occurs when the stent becomes partially dislodged from its balloon delivery system while crossing a resistant or tight lesion, or when the stent deforms and disengages from the balloon during withdrawal.

Management options include retrieval, deployment, or crushing of the lost stent (139, 144). Initial management generally begins with percutaneous retrieval. One common approach involves inflating the partially engaged balloon within the lost stent and carefully withdrawing the system into the guide catheter. Another option is the small-balloon technique, where a guidewire is advanced distal to the unexpanded stent, and a small balloon is positioned, inflated at low pressure, and gently pulled back to retrieve the stent into the guide catheter (145). If balloon inflation causes stent deformation that prevents guide catheter entry, the balloon, stent, and guide catheter may be removed together as a single unit (145).

If this method fails, assessment of stent-vessel size compatibility is necessary. When the stent size is appropriate for the vessel, deployment *in situ* can be considered (139, 144). In cases of size mismatch or poor positioning, other options include retrieval techniques similar to those used for complete stent loss, crushing with another stent, or advancing and redeploying the stent to a more suitable location (139, 144). The stent-crush technique involves deploying a second stent at the same site to compress the displaced stent. However, this should be avoided in critical segments, particularly the left main coronary artery, due to the risk of acute thrombosis or in-stent restenosis (139, 144). Adequate stent apposition is crucial to prevent thrombosis; therefore, the use of intravascular imaging such as OCT or IVUS is recommended to assess expansion and apposition (146). Stent advancement and deployment can be performed by slightly inflating the balloon to stabilize the stent, carefully advancing it to the target site, and then deploying it (145, 147).

### 8.2 Total stent loss

Management of total stent loss depends on whether the guidewire remains *in situ*. When the guidewire is still positioned across the lesion, the procedure begins with the small-balloon technique, similar to the approach used for partial stent loss. If this fails, stent-vessel sizing should be assessed. When the stent size is appropriate for the vessel, deployment at the current location can be considered (139, 144). If there is a size mismatch, stent advancement and redeployment using a balloon to reposition the dislodged stent to a more suitable site may be attempted (139, 144).

If the stent cannot be successfully deployed, snaring, guidewire twisting, or stent-crush techniques can be considered as alternative bailout strategies (139). The snare technique involves engaging the proximal end of the dislodged stent with a snare loop and withdrawing the captured stent into the guide sheath (139). In the twisting-guidewire technique, one or more additional guidewires are advanced through the stent struts and rotated together using a torquing device to entangle and retrieve the stent (121). If retrieval attempts are unsuccessful, the crush technique may be performed by deploying another stent at the same site to compress the dislodged stent against the vessel wall. In cases of total stent loss with guidewire loss, a new guidewire should first be advanced distally to secure vessel access. Subsequently, the snare technique should be attempted; if retrieval fails, the crush technique remains a reasonable alternative.

Extra-coronary stent dislodgement can also occur, with management determined by the location of the lost stent. When the stent migrates into large peripheral arteries, such as the iliac or common femoral artery, snaring or crushing with a peripheral stent may be appropriate (139, 148). In contrast, stent dislodgement into smaller branches below the common femoral artery is typically benign, and conservative management has been reported successful in such cases (139, 145, 149).

## 9 Air embolism

### 9.1 Description

Air embolism is an uncommon but serious complication of PCI that can result in abrupt vessel closure. The reported incidence ranges from 0.1% to 0.3% (150). Clinical manifestations vary from asymptomatic or transient ischemic symptoms to severe presentations, such as cardiogenic shock, malignant arrhythmias, or sudden death, particularly in cases of massive air embolism (150). Electrocardiographic findings in intracoronary air embolism include ST-segment elevation, nonspecific ST-T wave changes, T-wave inversion, atrioventricular block, and ventricular tachyarrhythmias (150). Evidence from *in vivo* studies have demonstrated that the volume of air introduced into the coronary circulation directly correlates with the degree of myocardial dysfunction and mortality in animal models (151). The most common cause of air embolism is inadequate catheter preparation, particularly incomplete aspiration of the guide or delivery system before device insertion. Other contributing factors include balloon rupture or leakage, prolonged negative suction, device exchanges through the guide catheter, deep intubation or rapid withdrawal of the guiding catheter, and leaky manifold or connector systems (150, 152). Diagnosis is typically established by direct angiographic visualization of round, mobile radiolucent bubbles within the coronary lumen. In more complex or static lesions where bubbles may be immobile or subtle, OCT can be used for confirmation (153).

### 9.2 Management strategies

Management focuses on prompt hemodynamic stabilization and oxygen therapy. Administration of 100% oxygen accelerates the reabsorption of intravascular air (150). In hemodynamically unstable patients, inotropic support or mechanical circulatory assistance should be initiated as needed. Treatment options include forceful injection of saline or blood, or using a guidewire to disrupt the air embolus, promoting dispersion and distal blood flow (150). Balloon aspiration may also be performed (150, 154). In cases where vasospasm is present or when air bubbles obstruct distal vessels or the microcirculation, leading to slow flow and myocardial blush, intracoronary adenosine can be administered to facilitate coronary blood flow (154).

## 10 Unintended stent deformation

### 10.1 Description

Unintended stent deformation (USD) refers to unplanned mechanical distortion of an implanted coronary stent, leading to deformation, fracture, or mechanical obstruction. If unrecognized or untreated, USD may result in stent thrombosis, loss of coronary access, and an increased risk of MACE (155, 156).

USD is frequently underrecognized because it may be angiographically occult. Intracoronary imaging is essential in aiding diagnosis. OCT, due to its high spatial resolution, is effective for detecting subtle deformation patterns, overlapping or diverging struts, abluminal wire position, and longitudinal stent deformation (155–158).

Mechanistically, USD most commonly results from accidental abluminal rewiring and guide catheter or guide-extension catheter collision with the stent edge, particularly during repeated rewiring and device manipulation in bifurcation PCI (155). In the OCTOBER trial substudy, USD was identified by OCT in 9.3% of bifurcation PCI cases and in 18.5% of left main bifurcation interventions; abluminal rewiring and guide catheter collision accounted for approximately 44% and 40% of cases, respectively (155). Stent design features, including reduced longitudinal strength in thin-strut platforms, especially in the setting of complex lesions including calcification, tortuosity, long and ostial disease, may further predispose to deformation (159). Importantly, untreated USD was associated with more than a twofold increase in the risk of MACE at 2 year, whereas treated USD was not associated with an increased risk of MACE (155).

### 10.2 Management strategies

Management of USD is guided by intracoronary imaging and includes high-pressure balloon post-dilation, focal optimization of the deformed segment, correction of abluminal rewiring, and additional stent implantation (155).

### 10.3 Preventive strategies

Preventive strategies emphasize proper bifurcation technique, routine verification of wire position, avoidance of deep guide catheter intubation, cautious use of guide-extension devices, and use of intracoronary imaging, particularly in complex PCI.

## 11 Coronary anatomical variations and PCI risk

Coronary anatomical variations contribute to procedural complexity and the risk of PCI-related complications. Variations in vessel size, tortuosity, angulation, calcification, and bifurcation geometry can significantly influence guide catheter engagement, guidewire manipulation, device deliverability, lesion preparation, and stent deployment (160). Complex anatomical features such as severe calcification, long or eccentric lesions, bifurcations, vessel tortuosity, and CTO increase procedural difficulty and predispose to acute vessel closure, coronary dissection, perforation, device entrapment, and stent loss (6, 11, 75–77, 79–81, 109). Careful pre-procedural anatomical assessment, appropriate lesion preparation, and intracoronary imaging-guided PCI are important for mitigating anatomy-predisposed complications and improving PCI outcomes.

## 12 Role of intracoronary imaging in PCI-related complications

Intracoronary imaging with IVUS and OCT plays an important role in guiding PCI in contemporary practice, from pre-intervention assessment and intraprocedural guidance to post-intervention evaluation. In the setting of PCI-related complications, these modalities aid in diagnosis, guide management, and potentially prevent adverse events by providing detailed assessment of vessel size, plaque morphology, and stent-vessel interaction beyond angiography alone (158).

IVUS, with deeper tissue penetration, is particularly useful for evaluating complex coronary anatomy and heavily calcified lesions, guiding lesion preparation, vessel sizing, and stent expansion. IVUS guidance has been associated with lower rates of major complications, including coronary perforation, especially in complex PCI and CTO interventions (83). IVUS is also effective for identifying PCI-related complications such as coronary dissection and intramural hematoma, intracoronary thrombus, coronary perforation, and retained or fractured intracoronary equipment (10, 115).

OCT provides higher spatial resolution using near-infrared light, enabling detailed visualization of stent malapposition, underexpansion, deformation or collapse, and edge dissection. OCT also assists in diagnosing PCI-related complications, including intracoronary thrombus and subtle vessel injury or air embolism (146, 153). However, due to the need for contrast injection and limited penetration depth, OCT should be used cautiously in the setting of active dissection, perforation, or advanced chronic kidney disease (16).

## 13 Failure to rescue after PCI-related complications

Failure to rescue (FTR), defined as death following a procedural complication, has emerged as an important quality metric in procedural outcomes (161). Data from the National Cardiovascular Data Registry, a large U.S. observational registry, demonstrated that although the overall incidence of PCI complications is low, in-hospital mortality approaches 20% among patients who experience a complication, compared with 1.3% in those without complications (3). Coronary artery perforation and coronary dissection were associated with high FTR rates, approximately 12.3% and 7%, respectively. These findings underscore the importance of preventive strategies, as well as timely recognition and management. The study also demonstrated a significant hospital-level variation in FTR, highlighting the influence of institutional factors such as catheterization laboratory protocols, complication detection pathways, availability of rescue tools, and team-based response systems (3). Understanding patient- and procedure-related risk factors, the pathophysiology of PCI complications, and evidence-based management strategies can support the development of standardized rescue protocols and postprocedural monitoring practices, ultimately improving procedural outcomes.

## 14 Conclusions

PCI has been increasingly performed with a lower incidence of complications. However, PCI-related complications still occur and can lead to adverse clinical outcomes. These include vessel closure, perforation, dissection, entrapment, stent loss, and embolization. Preprocedural and intraprocedural risk stratification, an understanding of the underlying mechanisms, and evidence-based management, along with the use of adjunctive tools such as intracoronary imaging, can mitigate the incidence and improve outcomes of PCI-related complications. An evidence-based approach is essential to achieve optimal short- and long-term results; however, due to the low incidence of many of these complications, current management strategies are often derived from case reports, observational studies, and expert opinion. Further large-scale studies utilizing clinical databases and artificial intelligence-guided risk stratification are warranted to enable individualized prediction, enhance procedural safety, and advance precision management aimed at reducing complications and improving patient outcomes.
